# The measurement and modification of hypoxia in colorectal cancer: overlooked but not forgotten

**DOI:** 10.1093/gastro/goac042

**Published:** 2022-08-25

**Authors:** Teddy Fletcher, Alex J Thompson, Hutan Ashrafian, Ara Darzi

**Affiliations:** Department of Surgery and Cancer, Queen Elizabeth the Queen Mother Wing, St Mary’s Hospital, Imperial College London, London, UK; The Hamlyn Centre, Institute of Global Health Innovation, Imperial College London, London, UK; Department of Surgery and Cancer, Queen Elizabeth the Queen Mother Wing, St Mary’s Hospital, Imperial College London, London, UK; Department of Surgery and Cancer, Queen Elizabeth the Queen Mother Wing, St Mary’s Hospital, Imperial College London, London, UK

**Keywords:** hypoxia, colorectal neoplasm, prognostication, neoadjuvant chemoradiotherapy

## Abstract

Tumour hypoxia is the inevitable consequence of a tumour’s rapid growth and disorganized, inefficient vasculature. The compensatory mechanisms employed by tumours, and indeed the absence of oxygen itself, hinder the ability of all treatment modalities. The clinical consequence is poorer overall survival, disease-free survival, and locoregional control. Recognizing this, clinicians have been attenuating the effect of hypoxia, primarily with hypoxic modification or with hypoxia-activated pro-drugs, and notable success has been demonstrated. However, in the case of colorectal cancer (CRC), there is a general paucity of knowledge and evidence surrounding the measurement and modification of hypoxia, and this is possibly due to the comparative inaccessibility of such tumours. We specifically review the role of hypoxia in CRC and focus on the current evidence for the existence of hypoxia in CRC, the majority of which originates from indirect positron emission topography imaging with hypoxia selective radiotracers; the evidence correlating CRC hypoxia with poorer oncological outcome, which is largely based on the measurement of hypoxia inducible factor in correlation with clinical outcome; the evidence of hypoxic modification in CRC, of which no direct evidence exists, but is reflected in a number of indirect markers; the prognostic and monitoring implications of accurate CRC hypoxia quantification and its potential in the field of precision oncology; and the present and future imaging tools and technologies being developed for the measurement of CRC hypoxia, including the use of blood-oxygen-level-dependent magnetic resonance imaging and diffuse reflectance spectroscopy.

## Introduction

Ever since Gray *et al.* [1] demonstrated the critical role of oxygen in the radiobiological process almost 70 years ago, researchers have been both fascinated and tormented in their efforts to measure and modify tumour hypoxia. Common to all solid tumours [2, 3], tumour hypoxia is the inevitable consequence of a tumour’s rapid growth, demanding metabolism, and disorganized, inefficient vasculature. The compensatory mechanisms employed by tumours, and indeed the absence of oxygen itself, hinder the ability of all treatment modalities, including radiotherapy, chemotherapy, and more recently, immunotherapy [4]. The clinical consequence of tumour hypoxia is therefore poor overall survival (OS), disease-free survival (DFS), and locoregional control [5]. Recognizing this, clinicians have been attenuating the effect of hypoxia, primarily with hypoxic modification or with hypoxia-activated pro-drugs. The largest meta-analysis addressing the effectiveness of such an approach demonstrated significant improvements in OS and locoregional control [6]. This was particularly true for head and neck cancers [7], and for cancers of the uterine cervix, bladder, and lung to lesser degrees. However, none of the included studies focused on colorectal tumours. Indeed, there is a general notable paucity of evidence pertaining to the measurement and modification of hypoxia in colorectal cancer (CRC).

There are several possible explanations for this. First, the most reliable method for the measurement of tumour oxygenation status is with Eppendorf electrodes, which are inserted directly into a tumour. The inaccessible nature of CRC thus makes such a measurement challenging. Additionally, the anatomical proximity of the rectum to the urinary bladder can complicate hypoxia assessment, and this is certainly true for positron emission tomography (PET) imaging. However, the studies included in Overgaard’s meta-analysis did not include measurement of hypoxia pre- or post-application of hypoxic modification—rather it was applied ‘blindly’, which weakens this theory since measurement of hypoxia was not a pre-requisite. Second, it could be that baseline CRC characteristics such as enhanced angiogenic activity [8] or different genetic or proteomic regulation mean that they are less prone to hypoxia, and the subsequent detrimental molecular adaptations, than other solid tumours. Third, it may simply be that hypoxic modification in colorectal tumours was overlooked because of the relative unimportance of radiotherapy in the management of CRC at the time these studies were performed.

The answer to this has important implications. The role of radiotherapy in rectal cancer is continually expanding as we enter the era of organ preservation and watch and wait following neoadjuvant chemoradiotherapy (nCRT). The presence and measurement of CRC hypoxia could therefore have critical implications for prognostication and response monitoring given its central role in chemoradiotherapy resistance. Below we briefly explain the importance of hypoxia in therapy resistance and how it can be measured and modified. We then examine the evidence for hypoxia specifically in CRC, whether its presence correlates with impaired therapy response and survival, and any evidence pertaining to its modification and consequent improved oncological outcome. We describe how the accurate quantification of hypoxia in CRC could greatly enhance prognostic capabilities and could be used for real-time chemoradiotherapy response monitoring, before finally outlining the future directions of CRC hypoxia measurement and its wider implications on surgical oncology.

## The role of hypoxia in the radiobiological process

**Figure 1.**
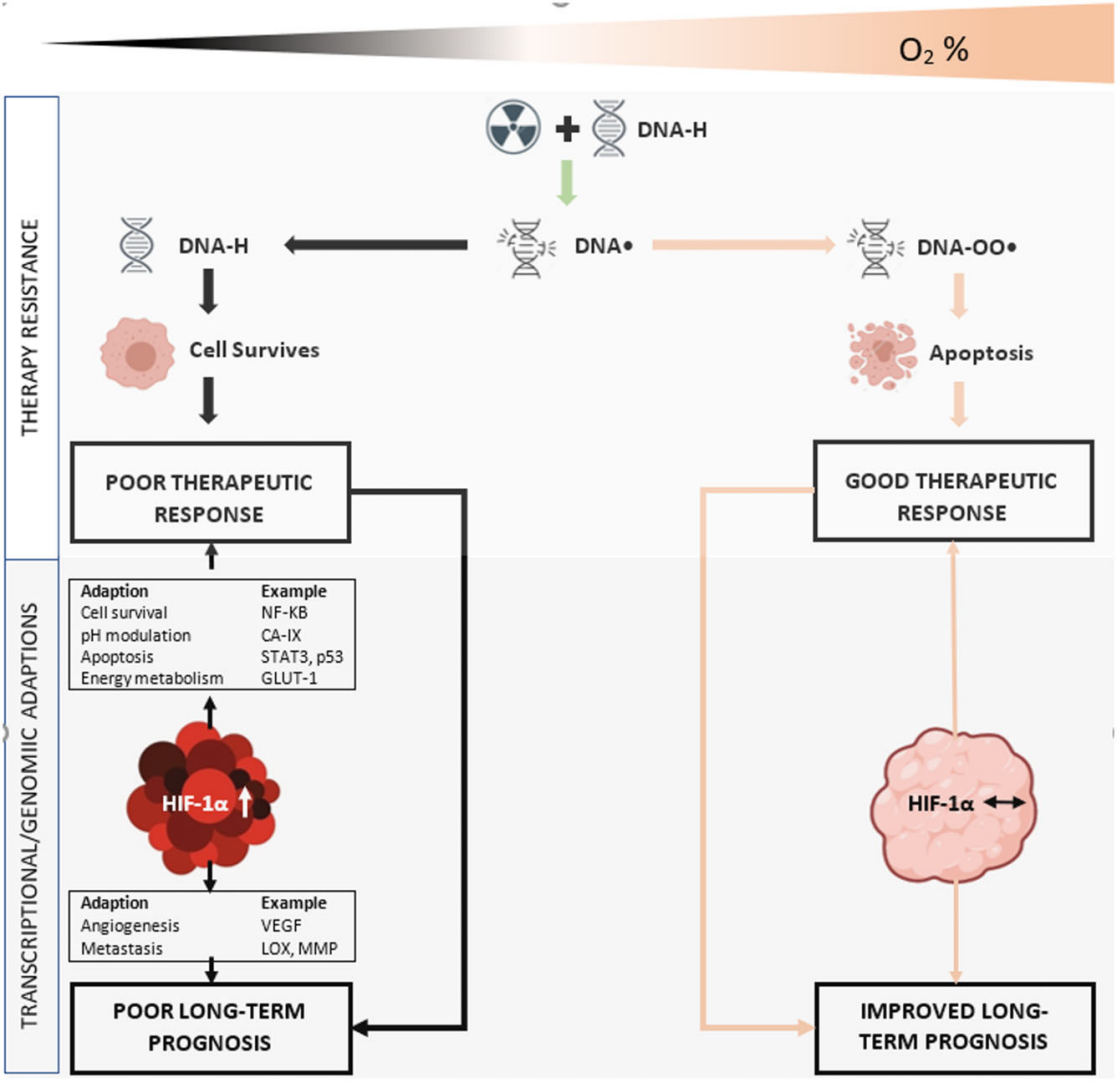
Schematic simplification highlighting the importance of oxygen in the radiobiological process and the hypoxia-induced transcriptional, genomic, and proteomic alterations leading to poor therapy response and poor overall survival. HIF, hypoxia inducible factor; GLUT-1, glucose trasporter-1; LOX, lysyl oxidase; MMP, matrix metalloproteinase; NF-κB, nuclear factor of kappa-light-chain-enhancer of activated B-cells; p53, tumour protein 53; STAT3, signal transducer and activator of transcription 3; VEGF, vascular endothelial growth factor; CA-IX, carbonic anhydrase 9. Ionizing radiation impacts directly with DNA, resulting in ionization damage, DNA•. This can easily be repaired to its original state (DNA-H), but in the presence of oxygen, a peroxy radical is formed (DNA-OO•), ‘fixing’ damage into a permanent irreparable state. Hypoxia results in the upregulation of HIF-1α, a master transcriptional protein with numerous downstream regulatory genomic and proteomic effects, leading to enhanced cell survival, invasion, metastasis, and consequent poor overall survival.

The role of tumour hypoxia in therapy resistance is well known. Cells situated over a certain distance (typically 100–150 µm) from a functional blood vessel are prone to chronic (>24 h) diffusion limited hypoxia [1]. Solid tumours are also prone to episodes of transient vessel occlusion leading to periods of acute hypoxia (<24 h). The result of these confounding mechanisms is a cyclical, heterogeneous map of tumour hypoxia, further complicated by varying rates of oxygen consumption between cellular components. The role and availability of oxygen in the radiobiological process are critical. Radiation induces apoptosis by directly or indirectly generating double-stranded DNA breaks. The oxygen fixation hypothesis describes how, in the presence of oxygen, ionizing radiation causes the oxidation of free radicals to DNA [9], thus causing irreparable damage. The irreversible nature of this oxidation process is vital: without it, cells can affect DNA repair and avert cell death (Figure 1). So important is this oxidative effect that 2.5–3.0 times the dose of ionizing radiation (IR) is required to achieve the same effect in the absence of oxygen—the so-called oxygen-enhancement ratio [10]. The presence of tumour hypoxia is not binary; rather it exists on a gradient of oxygen pressures. Radiobiological hypoxia, the level at which the effect of IR is limited in a manner described above, occurs at ≤3 mmHg (0.4%). However, other biological phenomena can occur at less severe levels of hypoxia and therefore anything below normoxic states can negatively affect therapy response [6].

The consequences of hypoxia on resistance extend beyond the aforementioned oxygen-induced DNA damage. Up/downregulation of >100 genes are known to be involved in a cell’s drive for survival and adaptation to hypoxic conditions. Central to their regulation are the master transcription factors, hypoxia inducible factors (HIFs) 1–3 [11, 12]. The far-reaching effects of HIFs 1–3 are beyond the scope of this review. However, it is important to note that the downstream regulation of a number of genes, exemplified in Figure 1, affect apoptosis, glycolysis, angiogenesis invasion, metastasis, and the cell cycle to enhance a cell’s survival capability. The differing degrees of hypoxia leading to these individual phenomena have been well researched and have been comprehensively reviewed elsewhere [13].

## Measuring and modifying hypoxia

The measurement of tumour hypoxia can be divided 3-fold. The first category is the direct quantification of the amount of oxygen in tissues using polarographic needle oxygen electrodes, now commonly referred to as Eppendorf electrodes. This methodology is accurate and enabled the fundamental link to be realized between tumour hypoxia and poor outcomes [14–17]. Unfortunately, the technique is invasive, user-dependent, impracticable for inaccessible tumours, and can be incapable of differentiating irrelevant necrosis from treatment-decisive severe hypoxia.

The second category is the use of hypoxic markers/tracers that reduce or bind to metabolic components under hypoxic conditions and can be identified on subsequent imaging (e.g. PET) or immunohistochemical staining (e.g. Pimonidazole staining). The most promising of these is PET imaging following injection of a radiotracer—e.g. ^18^F-Fluoromisonidazole (^18^F-FMISO) or ^18^F-fluoroazomycin-arabinofuranoside (^18^F-FAZA)—which diffuses into cells and, under hypoxic conditions only, binds to intracellular macromolecules. Notably, non-uptake of ^18^F-FMISO has been associated with better outcomes and OS in gliomas, head and neck, renal, and breast tumours (although not in rectal cancers due to non-specific uptake in the surrounding normoxic tissues) [18]. PET imaging has also been used to map tumour hypoxia, with consequent planning and application of intensity modulated radiation therapy (IMRT), in which higher doses of radiation are directed to hypoxic regions [19, 20]—so-called dose-painting.

The third category involves the indirect method of measuring biological processes that are known to affect or result from hypoxia, such as gene or protein expression (which are discussed below).

Methods for modifying hypoxia can be split into four key categories: (i) increasing the oxygen available, through the inhalation of hyperbaric or normobaric oxygen/carbogen; (ii) mimicking the role of oxygen in the radiobiological process with nitroimidazoles; (iii) selective destruction of hypoxic cells with hypoxia-activated pro-drugs such as tairapazamine; (iv) inhibiting isolated molecular/proteomic components, such as HIF-1α inhibitors; and (v) elimination of the oxygen-enhancement ratio effect with IMRT/radiotherapy dose-painting. Regarding approaches (i) and (ii), readers are referred to several comprehensive reviews assessing the practicalities and efficacies of each [21–26]. It should be noted that, despite showing early promise (particularly within the realm of head and neck cancers), neither approach (i) nor approach (ii) has been adopted. This is primarily due to the collateral tissue injury and toxicity inherent to these techniques. Additionally, their potential efficacy was largely undermined by the absence of a control (i.e. the presence of hypoxia was not known or measured prior to their application). Approaches (iii)–(v) encompass emergent novel approaches and are further detailed below.

### Hypoxia-activated pro-drugs

For reasons already detailed, the presence of hypoxia confers a significant survival advantage to the affected cell. It should also be noted that the presence of hypoxia significantly reduces the cellular proliferation rate [2]. Chemoradiotherapy primarily targets proliferating cells, consequently allowing surviving hypoxic cells to migrate to aerobic conditions prior to recommencing abnormal proliferation [27]. It is therefore hypothesized that directly targeting hypoxic cells could confer a significant treatment advantage. Hypoxia-activated pro-drugs (HAPs) are compounds that are reduced (by specific reductases) to their cytotoxic state only under hypoxic conditions. Several classes of HAPs have been developed, but the most important are Tirapazimine, PR-104, AQ49 (Banoxantrone), TH-302 (Evofosfamide), and SN30000. All these agents have demonstrated significant treatment advantage in both *in vitro* and *in vivo* studies [28, 29]. Most relevant to this review, Haynes *et al.* [30] demonstrated that the addition of Evofosfamide to 5-fluorouracil (5-FU) or CRT in colorectal xenograft tumours inhibited tumour growth and, significantly, the colorectal cancer-cell initiating (CC-IC) fraction*.* However, clinical trials on CRC patients have yet to be performed. Several of the aforementioned agents have been clinically trialled in other cancers, and although showing some promise, none has been adopted into clinical use. Unfortunately, the key barrier (drug toxicity aside) appears to be unchanged from the previous five decades: we still lack an effective way to quantify hypoxia, making it very difficult to stratify tumours according to their hypoxic status. Additionally, biomarkers to predict drug sensitivity are still required, since the drug efficacy is dependent on the presence of specific enzymes to complete the reduction reaction.

### HIF-1α inhibitors

Given the central role of HIFs 1–3 in the cellular response to hypoxia, it is perhaps unsurprising that a significant volume of research has been focused on attempts to inhibit these master transcription factors. Approaches can be broadly divided into the following three categories: (i) the direct inhibition of HIFs 1–3; (ii) the inhibition of downstream genetic and proteomic dependents affecting, for example, angiogenesis, apoptosis, glycolysis, and the DNA damage response; and (iii) the inhibition of upstream molecular components with a consequent knock-down effect on HIF production. These approaches have been reviewed conclusively elsewhere [31]. To date, there have been no approved drugs that directly inhibit HIFs 1–3 despite significant efforts to develop small-molecule inhibitors. This failure likely represents the extraordinarily complex up/downstream regulation of the HIF pathway, resulting in an incomplete understanding of tumour and inter-HIF signalling. Nonetheless, there have been some notable successes in upstream inhibition. This is most clearly exemplified by Irinotecan (a derivative of Camptothecin), licensed for the treatment of CRC in 1996 [32]. Its mechanism of action is inhibition of the TOP1 enzyme (an upstream regulator of HIF), thus leading to HIF-1α downregulation. Downstream inhibition of HIF-controlled components includes drugs such as vascular endothelial growth factor receptor inhibitors (e.g. bevacizumab) and these are discussed in more detail below. Although it is probably too pessimistic to state that the HIF transcription factors are ‘undruggable’, it is certainly fair to surmise that our understanding of the HIF pathway needs significant development before this approach becomes viable.

### IMRT/dose-painting

IMRT or dose-painting refers to the targeted delivery of intensified radiotherapy doses to hypoxic, and therefore comparatively radioresistant, regions of a tumour. Whilst entirely logical in theory, it remains practically problematic. The greatest barrier to this approach is the accurate identification of hypoxic tumour regions, which are both heterogeneous and cyclical in nature. Nonetheless, there are several papers reporting successful hypoxia mapping with PET imaging (in non-CRCs) with consequent IMRT [19, 20]. With the emergence of ever-more sophisticated imaging techniques (described below), this is likely to be a rapidly expanding field.

## Are colorectal tumours hypoxic?

**Figure 2.**
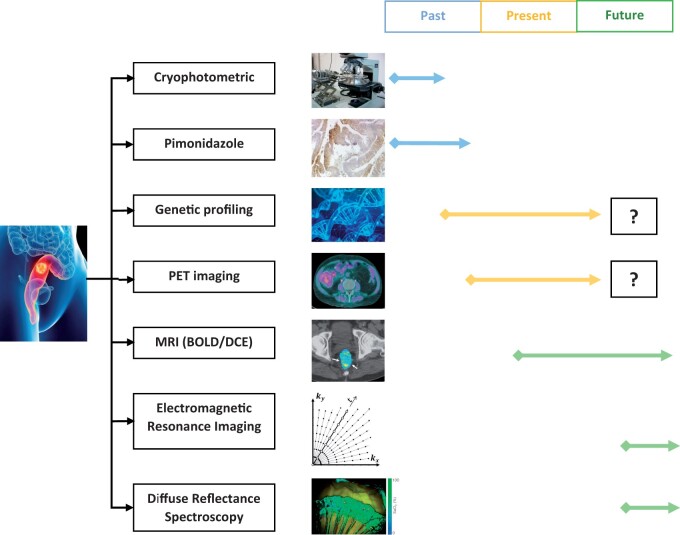
Methods used in the past, present, and (potentially) future for the measurement of colorectal cancer hypoxia. Due to inherent difficulties, the future of genetic profiling and positron emission tomography imaging for hypoxia assessment is in doubt. The most promising imaging techniques and technologies for the future are blood-oxygen-level-dependent and dynamic contrast-enhanced magnetic resonance imaging, electromagnetic resonance imaging, and diffuse reflectance spectroscopy.

**Figure 3.**
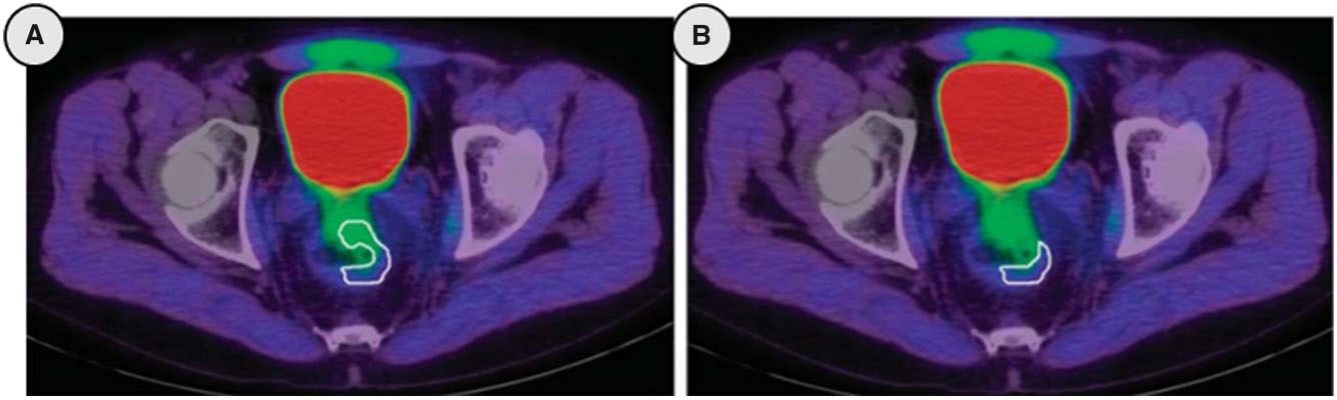
Positron emission tomography imaging with ^18^F-fluoroazomycin-arabinofuranoside hypoxic-specific radiotracer of a rectal tumour. The white lines represent (a) the volume of the entire tumour, with significant backscattering from the urinary bladder; and (b) the remaining volume of the tumour that can be observed due to the backscattering effect.

No studies to date have used Eppendorf electrodes to measure CRC oxygenation status and thus there is no direct evidence of CRC hypoxia. Therefore, all available knowledge stems from indirect methods (Figure 2) and PET imaging following the injection of hypoxic-specific tracers forms much of this evidence—although only a small number of studies exist [33–36]. Put simply, this technique works by injecting the radiotracer ∼2 h prior to PET imaging. In theory the tracer is then selectively retained in hypoxic cells and cleared from normoxic cells, although in reality there is often considerable overlap between hypoxic retention and normoxic clearance. The standard uptake value (SUV) is calculated, which reflects uptake relative to patient body weight and administered dose. Ratios between maximal (SUV_max_) tumour uptake and mean (SUV_mean_) muscle (T/M) and intestinal wall (T/I) uptake are calculated, allowing relative quantification of hypoxia. In 2015 Havelund *et al.* [36], using the radiotracer ^18^F-FAZA, demonstrated a significantly higher radiotracer uptake in rectal cancer (RC) than in the surrounding muscle and intestinal wall, thus confirming that hypoxia exists in RCs. These findings are corroborated by additional studies using both ^18^F-FMISO [34] and ^60^Cu-diacetyl-bis (N4-methylthiosemicarbazone) (^60^Cu-ATSM) [35], which demonstrated significantly greater uptake of the hypoxic-specific tracer in 9 out of 10 and 14 out of 17 RCs, respectively. Further, the same authors observed that SUV and T/M ratios were comparable to those reported in ^18^F-FAZA studies of the head and neck [37, 38], where hypoxia is known to have a detrimental impact on therapy response and outcome. While these studies appear to confirm the presence of rectal tumour hypoxia, it should be noted that this technique is vulnerable to significant scattering effect from the urinary bladder (Figure 3), meaning only part of the tumour can be assessed. Additionally, the heterogeneous nature of tumour hypoxia cannot be fully appreciated from the resulting images, thus limiting the usefulness of this technique in planning selective high-dose radiotherapy.

However, such heterogeneity was demonstrated by Wendling *et al.* [39] using a crayophotometric method, which involves the immediate freezing of tumour biopsy samples in nitrogen oxide; sectioning of each biopsy into 15-µm slices; assessment under a microscope cryostat for the photometric detection of single red blood cells; and consequent calculation of HbO_2_ saturation from observed spectra. First, it was demonstrated that the mean HbO_2_ saturation was 42% in the tumour and 80% in healthy rectal wall biopsies, thus confirming tumour hypoxia. Second, HbO_2_ saturation ranges of 0%–100% were found within different areas of the same rectal tumour, thus confirming significant intra-tumoural heterogeneity. Third, and perhaps most importantly, there was significant inter-individual heterogeneity for biopsies taken in the same location for tumours of the same grade, size, and histological stage. Given the importance of oxygen in the radiobiological process, it is therefore reasonable to conclude that rectal tumours of the same stage have heterogenous radiosensitivities, thus accounting for the observed variable response to radiotherapy.

Further evidence of colorectal tumour hypoxia has been gained with pimonidazole, an exogenous hypoxia marker that selectively binds to intracellular macromolecules at low oxygen pressures (<10 mmHg). By taking several left colonic or rectal tumour biopsies several hours following pimonidazole intravenous administration, Goethals *et al.* [40] demonstrated that the proportion of hypoxic cells ranged between 2.2% and 37.8% (median 16.7%) and once again confirmed the heterogenous nature of the observed hypoxia. These results are comparable to the levels of pimonidazole staining seen in studies of colorectal liver metastasis [41] and cancers of the head and neck [42, 43], uterine cervix [44], and bladder [45].

The final method by which the presence of CRC hypoxia has been confirmed is through the measurement of endogenous genetic, proteomic, and molecular markers of hypoxia. The best-known of these is HIF-1α, a master transcriptional protein that is stabilized/upregulated in hypoxic conditions and has numerous measurable downstream regulatory genomic and proteomic effects. This ultimately leads to enhanced cell survival (NF-κB, p53, STAT3), angiogenesis (vascular endothelial growth factor [VEGF], epidermal growth factor receptor [EGFR]), and metastasis (LOX, MMP). There is a wealth of evidence attempting to correlate the expression of these and other molecular markers with poor therapy response and OS in CRC, and these are discussed below. However, it should be noted that this is a rather indirect assessment of hypoxia and the exact up/downregulation of HIF-1α, and its downstream regulatory effects remain incompletely understood and have not been adopted into routine clinical use. Indeed, attempts to correlate markers such as carbonic anhydrase 9 (CX IX), VEGF, EGFR, and GLUT-1 with pimonidazole staining have been unsuccessful [40, 41], thus reflecting the variable and inaccurate expression of such markers.

It therefore seems reasonable to surmise that, although colorectal tumour hypoxia has not been directly measured, there is encouraging indirect evidence to suggest that it exists in a similar degree and distribution to other solid tumours, and is therefore likely to have a similarly detrimental impact on therapy response, locoregional control, and OS.

## Does colorectal tumour hypoxia correlate with poor response to therapy and worse clinical outcome?

Almost all the evidence associating CRC hypoxia with impaired clinical outcome originates from studies focusing on the measurement of the above-mentioned endogenous markers of hypoxia, in combination with the development of more comprehensive genetic assays and panels that analyse a large number of genes simultaneously. Unfortunately, the published studies have focused on differing end points (e.g. pathological complete response [pCR], tumour regression grade, tumour downstaging, OS, DFS), timings of response assessment, and regimes and types of chemoradiation, making it difficult to draw meaningful comparisons between studies. Additionally, the technique is exposed to notable inaccuracy due to significant intra-tumoural heterogeneity, meaning that a single, or serial, pretreatment biopsy(ies) is/are likely to be futile, since the same tumour could harbour different genetic mutations in a neighbouring, but unbiopsied, area.

Nonetheless, a number of hypoxia-reflecting genetic/proteomic/transcriptional markers have been shown to predict therapy response and outcome in CRC. Perhaps unsurprisingly, the most significant of these is HIF-1α. In 2013, Chen *et al.* [46] performed a meta-analysis of 23 studies comprising 2,984 patients. The results indicated that overexpressed HIF-1α (and HIF-2α) was associated with a statistically significant poorer outcome, including OS and DFS. However, subgroup analysis indicated that this was only the case in Asian, and not European, populations (although this was explained by the smaller European study sizes leading to insufficient power to detect significant differences). Additionally, a number of studies have examined the link between HIF-1α and tumour response/regression and the occurrence of pCR following nCRT. In a small study of 40 patients, Toiyama *et al.* [47] demonstrated that responders (tumour regression of >2 pathological grades) to nCRT demonstrated significantly lower levels of HIF-1α (and VEGF and EGFR) gene expression on pretreatment biopsies than non-responders (tumour regression of ≤1 pathological grades). However, in a similarly sized study (*n *=* *50), Shioya *et al.* [48] found no correlation between HIF-1α expression and pathological grading or pCR rates following preoperative radiotherapy combined with hyperthermo-chemoradiotherapy. This stance is further supported by Havelund *et al.* [49], who found that HIF-1α (and GLUT-1) was not associated with a difference in tumour response post nCRT when assessed using the Mandard Tumour Regression Grading System (TRG). Such discrepancies serve to highlight the problematic heterogeneity (e.g. tumour downstaging or tumour regression) encountered in this field when attempting to draw conclusions, thus explaining why HIF-1α is, to date, not used as a clinically useful prognostic biomarker in RC.

Other studies have focused on up/downregulation of downstream genetic or proteomic markers dependent on HIF-1α. High levels of VEGF, EGFR, CA-IX, and GLUT-1 detected through immunohistochemical staining have all been associated with worse OS and DFS [47, 50, 51]. Further, Giralt *et al.* [52] demonstrated that positive expression of EGFR at pre-nCRT biopsy was associated with a failure to achieve pCR. However, it should be acknowledged that the precise genetic and molecular interactivity between HIF-1α and the above-mentioned gene expression profiles is incompletely understood, and, as such, using these as surrogate markers to assess the impact of hypoxia on outcome in RC is likely to be inaccurate and over-reliant on assumed correlations. Once again, these downstream markers are yet to be incorporated into clinical use.

More recently, researchers have been analysing multiple genes simultaneously to develop ‘hypoxic signatures’ or ‘hypoxic scores’. Such an approach is neatly exemplified by Derkevel *et al.* [53], who examined for gene expression alterations in colorectal cells grown in chronically hypoxic conditions. The expression pattern was then associated with the clinical outcome in >200 patients with CRC, and from this a six-gene Colon Cancer Hypoxia Score (CCHS) was developed. The 3-year DFS rate was significantly higher in those with a low, rather than high, CCHS (77.3% vs 46.4%, respectively; *P *=* *0.006). However, this score, along with other similar predictive scores and nomograms [54] was designed for prognostic purposes and have not been used for nCRT response prediction, and like the above genetic expressions of hypoxia, they have not been clinically used because of inherent inaccuracies.

Finally, there is only one small pilot study using PET imaging with hypoxia selective tracers to assess the impact of CRC hypoxia on nCRT response and clinical outcome. Using ^60^Cu-ATSM-PET, Dietz *et al.* [35] imaged 19 patients before nCRT for RC. Whilst the authors recognize the study was underpowered, their results suggested both worse OS and DFS in hypoxic tumours, as well as a poorer response (measured by tumour downstaging) to nCRT in the hypoxic group.

In sum, the inherent difficulties of measuring CRC tumour hypoxia through direct methods have hampered attempts to associate its presence with poor response to therapy and oncological outcome. However, despite the heterogeneous nature of the studies associating HIF and its downstream genetic dependencies, there does appear to be a probable association between HIF expression and poor outcome and response. A technology or device that could measure CRC hypoxia more directly and accurately would be of critical value in validating this correlation.

## Can CRC hypoxia be modified for improved therapy response and clinical outcome?

There are no studies that directly assess the effect of hypoxic modification on human CRC. The likely reason for this is the relatively limited role of radiotherapy in the management of RC at the end of the twentieth century—the period in which most of the studies on hypoxia modification were performed. Since the vast majority employed the application of enhanced oxygenation or administration of nitroimidazoles to enhance the effect of radiotherapy, CRC was unlikely to have been considered a viable candidate for modification. However, there are some methodologies and indirect lines of evidence that may reflect successful hypoxic modification in CRC.

The first of these is the advantageous use of hyperthermia (HT) in conjunction with nCRT. HT refers to the heating of a tumour and surrounding tissues to ∼42.5°C. It can be achieved externally using radiofrequency capacitive heating devices, internally using intraluminal heating electrodes, or through whole-body HT under sedation. HT is a compelling radiosensitizer and, when compared with radiotherapy alone, has been shown to significantly improve locoregional control and OS in cancers at several sites—including the head and neck, bladder, chest wall, cervix, rectum, and skin [55]. The evidence in support of hyperthermia in RC, compiled by Ohno *et al.* [56], consists mainly of small non-randomized trials or case series. However, there are a few exceptions worth noting here. Perhaps most significantly, Van der Zee *et al.* [57] performed a prospective, randomized trial of 258 patients comparing HT with radiotherapy vs radiotherapy alone for advanced pelvic cancers, including tumours of the rectum, bladder, and cervix. The radiotherapy plus HT group demonstrated superior complete response rates (55% vs 39%, *P *<* *0.001), longer duration of local control and greater 3-year survival (51% vs 27% *P *<* *0.001). Additional evidence was provided by Berdov *et al.* [58]*,* who used a 915-MHz microwave to create HT in conjunction with radiotherapy. In T4 tumours, pCR and significant regression rates were 16.1% and 53.6%, respectively, compared to 1.7% and 33.9% in the control group (radiotherapy only). Other randomized trials demonstrate the advantage of HT plus CRT over surgery alone [59, 60], but these are obviously less helpful in distinguishing the additional benefit of HT on top of CRT.

Relatively little is known about how hyperthermia potentiates the effects of radiotherapy. It is thought that the heat primarily disrupts a cell’s ability to repair radiation-induced DNA damage [61]. The other commonly offered explanation is that the additional heat improves blood supply and flow to the tumour, thus diminishing the negative impact of hypoxia on radiosensitivity [62], and hence the inclusion here of hyperthermia as an indirect method of hypoxic modification. Indeed, Song *et al.* [63] demonstrated that mild hyperthermia can lead to improved and sustained tumour oxygenation for ≤2 days and may be a more effective radiosensitizer than both carbogen breathing and nicotinamide. However, this stance is not supported by both Kelleher *et al.* [64] and Vaupel *et al.* [65], who argue that any improvement in oxygenation is transient and not significant enough to radiosensitize a tumour. Nonetheless, it remains true that hypoxic cells *in vitro* are far more sensitive to HT than normoxic cells [66, 67]. Importantly, under optimal nutrient conditions, acute hypoxia does not affect the radiosensitivity of cells. On the contrary, chronic hypoxia, which leads to the associated genetic and molecular changes (which result in changes in cellular pH and energy metabolism for example), strongly affects the efficacy of HT [68]—and it therefore seems logical to conclude that it is these underlying cellular changes, rather than reduced oxygenation per se, that affect HT sensitivity. Thus, HT as a ‘hypoxia modifier’, although relevant, appears tenuous. In any case, in Europe, HT has remained in the research domain, and it is only Japan and China, who invested heavily in the necessary infrastructure, who continue to investigate its use.

The second indirect maker of hypoxic modification in CRC is the presence and correction of preoperative anaemia. There are numerous studies evidencing the detrimental impact of preoperative and pretreatment anaemia on tumour response to nCRT [69–72], occurrence of pCR [73], locoregional control [74], DFS, and OS [75]. For example, Lee *et al.* [70] reported that a haemoglobin concentration of ≥9 g/dL resulted in TRG stages 3–4 in 29%, compared with 0% if the Hb was <9 g/dL (*P *<* *0.001). In a meta-analysis of 3,588 patients, Wilson *et al.* [75] found that preoperative anaemia was significantly associated with OS and DFS in rectal cancer—although they did recognize the high level of heterogeneity between the included studies.

The question is therefore less ‘if’ and more ‘why’ anaemia does have a deleterious effect on response and outcome. Perhaps the most obvious answer is that the reduced oxygen-carrying capacity of the blood exacerbates tumour hypoxia. Indeed, reversing anaemia by normalizing the haemoglobin does lead to enhanced tumour oxygenation in a clinical setting [76, 77]. Additionally, high levels of circulating angiogenic factors (VEGF)—a known consequence of tumour hypoxia—have been positively associated with anaemia in the presence of solid cancers [78]. Further still, anaemia is an established predictive factor for radiosensitivity in a number of other tumours such as those of the uterine cervix and head and neck [79, 80].

However, this apparently neat association is complicated somewhat when we consider the underlying cause of anaemia in CRC. First, the friable malignant mucosa of a tumour bleeds and it is therefore reasonable to assume that the larger the tumour, the greater the bleeding—this assumption has in fact been proved by both Väyrynen *et al.* [81] and Khan *et al.* [73], who correlated severity of anaemia with tumour length. Therefore, anaemia may simply reflect increased size, stage, and disease severity, and hence poorer OS. On this note, it should be recognized that none of the studies referenced above has prospectively demonstrated the association between anaemia and cancer-specific outcome. The other important cause of anaemia in CRC is systemic inflammation, commonly referred to as an ‘anaemia of chronic disease’, thus explaining the anaemia associated with other non-GI cancers and inflammatory conditions such as rheumatoid arthritis. The cytokine-driven inflammatory state interferes with the absorption and uptake of iron due an IL-6-influenced expression of hepcidin, which therefore results in an iron-deficient, but often normocytic, anaemia. Thus, anaemia is often a reflection of a tumour-driven, cytokine-mediated, systemic inflammatory response, therefore explaining the observed inverse correlation between severity of anaemia and C-reactive protein and the modified Glasgow Prognostic Score (mGPS) for inflammation [81]. It is likely that this inflammation has an important and, as yet, under-appreciated impact on radiosensitivity and oncological outcome. Indeed, the observation that the radiosensitivity of a tumour is related to the immunocompetence of the host [82] has prompted significant interest in the potential of immune modulation and use of immunotherapy in RC to improve radiosensitivity.

The relative importance of these two differing theories (of why anaemia associates with worse outcome)—hypoxia-enhancing or inflammatory-reflecting—is not known. However, if anaemia truly were hypoxia-enhancing, one could logically hypothesize that correcting it in the pretreatment phase would dissolve its adverse effect. Unfortunately, it has not been possible to test such a hypothesis, not least because almost every method of correcting anaemia has an unintended detrimental consequence: perioperative allogenic blood transfusions have been linked to increased recurrence rates and poor oncological outcome in CRC [83, 84]; the administration of erythropoietin (EPO) increases mortality and worsens OS [85]; and even iron transfusions, which are currently favoured for their superiority over oral supplementation [86] and ability to reduce the requirement for allogenic transfusion [87], have had their safety questioned over concerns that increasing the availability of iron may in fact support tumour growth and metastatic potential [88]. Thus, as far as the authors are aware, there are no studies assessing whether the pretreatment correction of anaemia improves oncological outcome.

The third indirect marker of hypoxia modification in CRC is the use of anti-angiogenic therapies targeting VEGF. If we accept that CR tumours are indeed hypoxic, the use of anti-angiogenic therapies such as bevacizumab (a humanized monoclonal antibody targeting VEGF) seems incongruous. However, it should be realized that the angiogenesis associated with malignant tumours is extremely abnormal. The underlying pathogenesis and mechanisms of malignant angiogenesis have been covered in detail elsewhere [89]. Briefly, the typical observation is one of torturous, irregular networks of hyperpermeable blood vessels, which remain in a permanent state of vasodilation due to the absence of smooth muscle formation and vasoconstrictive mechanisms [90–92]. Therefore, the role of anti-angiogenic therapies is in fact to reverse such abnormality and restore normal angiogenic function, and consequently improve tumour hypoxia. The efficacy of bevacizumab, and other anti-VEGF agents, is mixed. After initially promising results from two meta-analyses for metastatic and late-stage CRC [93, 94], it became apparent that it caused a small decrease in OS in earlier tumours [95], resulted in an increased rate of relapse on cessation [96], and led to tumour rebound—in which a more aggressive phenotype appeared after initial regression [97]. As such, bevacizumab is only licensed as a second-line treatment for metastatic CRC [98].

It is it therefore reasonable to conclude that, in the absence of evidence pertaining to direct hypoxia modification in CRC, it remains unknown whether CRC hypoxia can be modified for improved therapy response and oncological outcome. However, the above-discussed indirect markers of hyperthermia and anaemia, and to a lesser extent bevacizumab, certainly allow a degree of logical expectation that supports ongoing investigation.

## The true potential of quantifying CRC tumour hypoxia: advanced prognostication, real-time response monitoring, and precision oncology

Neoadjuvant chemoradiation therapy (nCRT), once of modest impact, is changing the way we manage RC [99]. Following Habre-Gamma’s pioneering work [100], there is great international interest in the phenomenon of pCR. Occurring in 15%–30% of RCs exposed to nCRT [101], pCR describes the finding of no residual tumour on histopathological analysis following surgical resection. The requirement to proceed with surgery in this patient subset has been questioned and is the focus of two large ongoing randomized trials—STARTREC [102] and TRIGGER [103]—investigating the safety and efficacy of ‘local excision with organ preservation’ and ‘watch and wait’ policies, respectively, following nCRT. The lure of major rectal surgery avoidance is strong, particularly for those with risk-enhancing co-morbidities. However, barriers remain to endorsement of this strategy by key health bodies [104]. First, it should be recognized that a significant proportion (20%) of tumours progress, or fail to regress, in spite of nCRT, meaning that any delay in proceeding with surgery whilst nCRT is completed may paradoxically increase the likelihood of distant metastasis. Second, success has been recorded for the early T1/T2N0 tumours, a group not normally offered nCRT. There is therefore a moral disquiet in offering nCRT to a patient with a good prognosis if the tumour is surgically resected. Third, the timing of reassessment following nCRT is arbitrary and disputed: whilst the tumour may still be present at 8 weeks, it may not be at 12. Thus, the requirement is clear: patient selection and prediction of pCR are paramount if we are to avoid undesirable outcomes and unnecessary delay from this novel strategy, particularly for patients for whom surgery is inevitable. Predictive nomograms [105] using patient and tumour features (such as circumferential resection margin involvement and distance from the anal verge) have been used to predict nCRT responses with promising, and yet inadequate, accuracy. Therefore, for an accurate prediction, a deeper knowledge of the tumour microenvironment and mechanism of chemoradiation resistance is required and a detailed knowledge of tumour hypoxia has the potential to greatly expand our predictive capabilities. Since the resulting transcriptional, proteomic, and genomic changes occur at different degrees of hypoxia, it is not enough to merely identify the presence or absence of tumour hypoxia, and accurate quantification is required to elicit the full prognostic potential.

**Figure 4.**
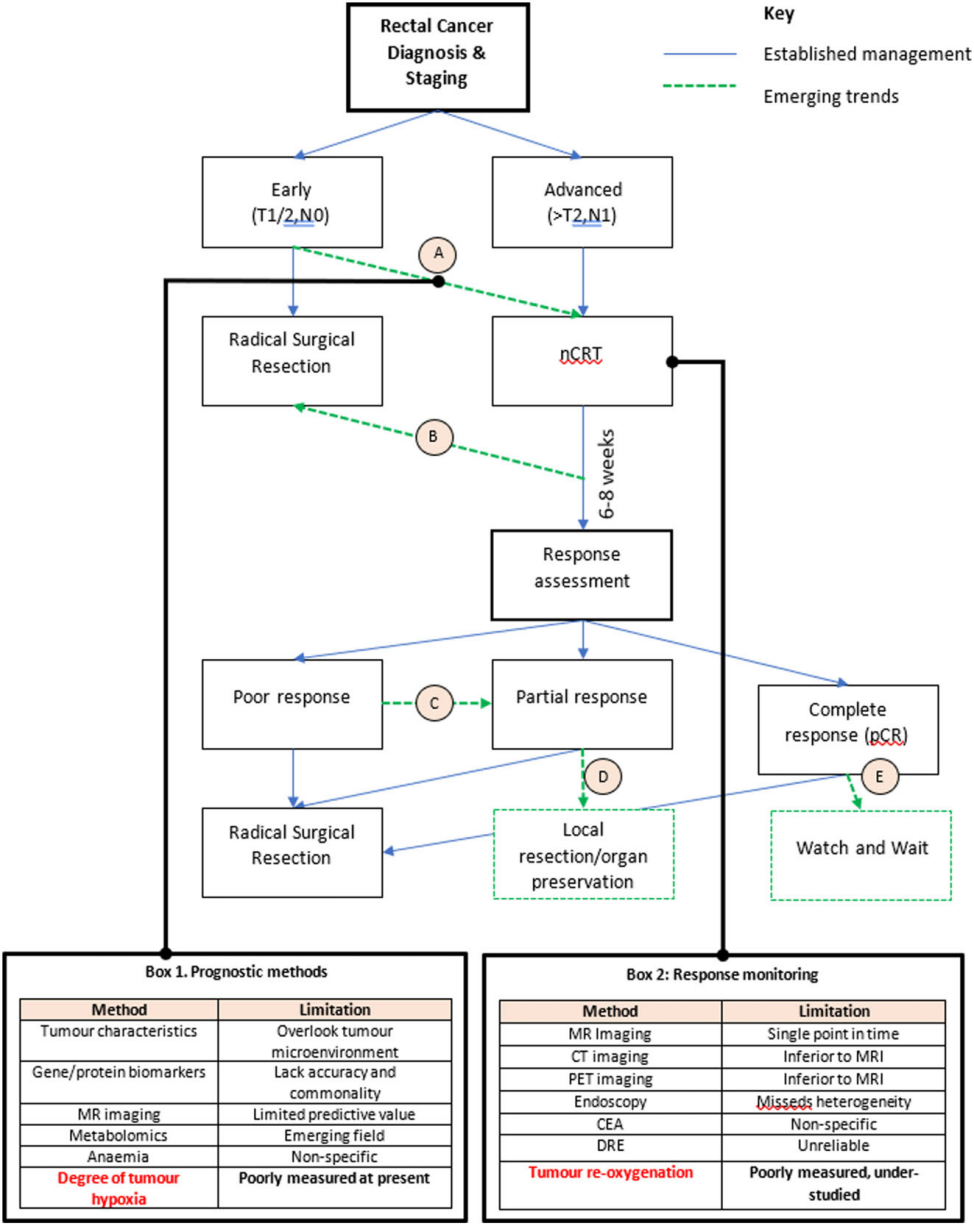
Simplified management algorithm of rectal cancer, highlighting the requirement for advanced prognostication and response monitoring in accordance with emerging trends. Advanced prognostication (A) would identify early tumours (not normally offered neoadjuvant chemoradiotherapy), which may respond to enable organ preservation (D) or watch and wait (E), and conversely identify advanced tumours that may not respond to neoadjuvant chemoradiotherapy and thus require immediate surgery. Continuous response monitoring would facilitate early identification of failure to respond and the need to proceed with surgery (B), as well as allowing time for a poor (C) or partial (D) responder to respond, thus avoiding major resection. Boxes (1) and (2) summarize the current methods and associated limitations of current prognostication and response monitoring, and highlight the potential role of tumour hypoxia assessment

Additionally, an ability to accurately monitor response to nCRT would enable a dynamic decision process and personalized tailoring of therapy (Figure 4). Whilst magnetic resonance imaging (MRI) result remains the gold standard for response assessment, it suffers from a significant lack of dynamism: it is performed at, somewhat arbitrarily, a single point in time, usually 6–8 weeks following nCRT. As discussed above, it is likely that some of these tumours would continue to regress, but the multidisciplinary team is often left with a binary decision: progress with surgery or deferral of surgery (watch and wait). Whilst repeat MRI is possible, it is time-consuming, costly, resource-intensive, and rarely done in practice. Therefore, an ability to measure the re-oxygenation of a tumour in response to a course of fractioned radiotherapy—which occurs through a process of reduced oxygen metabolism, improved circulation, tumour shrinkage, and migration of cells from hypoxic to aerobic conditions [27]—could enable real-time response monitoring of rectal tumours to nCRT, thereby potentially heralding an era of precision oncology in the management of CRC.

## Future directions of hypoxia assessment in CRC

A greater understanding of hypoxia in CRC could enable advanced prognostication—including the prediction of pCR; the application and accurate assessment of hypoxic modification; the use of IMRT, which delivers higher doses of radiotherapy to radioresistant areas of a tumour; and, perhaps most importantly, continuous tumour response monitoring by measuring the changing levels of hypoxia. However, to enable this understanding, it is very clear that new methods and technologies are required to accurately measure and map tumour hypoxia in CRC. Indeed, there are several ongoing exciting developments in this field (Figure 2). In a single-centre, non-randomized imaging study, Goh and colleagues are investigating the potential of blood-oxygen-level-dependent and dynamic contrast-enhanced (DCI) MRI to detect CRC hypoxia [106]. Both imaging techniques provide information on tumour dynamics and perfusion, but whilst they have shown promise, they provide qualitative, rather than the required quantitative, information on hypoxia. Concurrently, there is interest in the use of electronic paramagnetic resonance imaging for a quantitative readout of hypoxia, but significant improvements to readout technology is required [107] and this technology is not specific to CRC. Finally, diffuse reflectance spectroscopy (DRS) is also being investigated as a technique for *in vivo* measurement of RC hypoxia. DRS represents a promising technology with a proven ability to assess tissue oxygenation [108] and an evolving ability for carcinogenic assessment [109], which can be deployed using minimally invasive tools. It seems logical that a DRS probe could either be delivered via an endoscope for regular assessment of tumour hypoxia or, more ambitiously, as an implanted device to give real-time, continuous feedback on cyclical and heterogenous hypoxia. Thus, notwithstanding the challenges that remain (including issues relating to the effect of space and pressure at the probe–tissue interface [110]), DRS holds very real potential to enhance our understanding and management of tumour hypoxia in CRC.

## Conclusions

Our knowledge of CRC hypoxia remains somewhat limited, particularly in relation to other cancers. The evidence for the existence of hypoxia in CRC is encouraging but lacks confirmation from direct measurement. The mixed evidence pertaining to the effect of CRC hypoxia on therapy response and oncological outcome is based almost entirely on the genetic expression of endogenous markers of hypoxia—which are themselves variable and incompletely understood. Finally, there appears to be no evidence to date that CRC can be directly modified or targeted for the enhancement of oncological outcome. However, there are several surrogate markers which imply that CRC hypoxia has the potential for modification. There is therefore a clear and critical need for a device or methodology that can accurately measure CRC hypoxia to enable advanced prognostication, tumour response monitoring, and the application of hypoxic modification—and indeed, such work is underway with the development of new imaging and sensing tools.

## Authors' Contributions

All authors contributed to the review ideation and conception. The literature search, material preparation, and analysis were performed by T.F. The first draft of the manuscript was written by T.F., which was critically revised by A.T., H.A., and A.D. All authors read and approved the final manuscript.

## Funding

This work was supported by the National Institute for Health Research (NIHR) Imperial Biomedical Research Centre (BRC). The views expressed in this publication are those of the authors and not necessarily those of the National Health Service (NHS), the NIHR, or the Department of Health (DH).
